# Early Erythrocyte Methotrexate Polyglutamate-4 Predicts Treatment Response and Is Influenced by GGH 401C>T Genotype in Patients with Rheumatoid Arthritis

**DOI:** 10.5152/ArchRheumatol.2026.25173

**Published:** 2026-03-03

**Authors:** Miho Takahashi, Soshi Takahashi, Ayano Mori, Saori Hatachi, Toshiharu Saito, Masakazu Shinohara, Shunichi Kumagai

**Affiliations:** 1 The Shinko Institute for Medical Research, Shinko Hospital, Kobe, Japan; 2 The Center for Rheumatic Disease, Shinko Hospital, Kobe, Japan; 3 The Integrated Center for Mass Spectrometry, Kobe University Graduate School of Medicine, Kobe, Japan; 4 Division of Molecular Epidemiology, Department of Future Medical Sciences, Kobe University Graduate School of Medicine, Kobe, Japan

**Keywords:** Methotrexate, methotrexate polyglutamate, rheumatoid arthritis, single-nucleotide polymorphism

## Abstract

**Background/Aims:**

This study aimed to examine the ability of methotrexate polyglutamate (MTXPG) concentrations in erythrocytes to predict methotrexate (MTX) treatment response and evaluate the association between MTXPG concentrations and single nucleotide polymorphisms (SNPs) involved in MTX metabolism.

**Materials and Methods:**

Methotrexate polyglutamate (MTXPG) concentrations in erythrocytes were measured in 76 MTX-naive patients utilizing liquid chromatography-tandem mass spectrometry. Real-time polymerase chain reaction was conducted to identify solute carrier family 19 member 1, folylpolyglutamate synthase, and gamma-glutamyl hydrolase (GGH) genotypes. Associations between MTXPG concentrations and MTX treatment response and between MTXPG concentrations and SNPs were analyzed.

**Results:**

This study identified 67 MTX responders and nine non-responders at 24 weeks. MTXPG4 concentrations at four weeks were significantly greater in responders than in non-responders (*P* < .01). Receiver-operating characteristic analyses for detecting MTX responders at 24 weeks using MTXPG4 at four weeks revealed an area under the curve of 0.77 at a cut-off level of 0.46 nmol/L. The rate of achieving MTXPG4 of ≥0.46 nmol/L at four weeks was significantly higher in patients with CC genotype than those with CT and TT genotypes in GGH 401C>T (*P* = .032). The adjusted odds ratio of achieving MTXPG4 of ≥0.46 nmol/L at four weeks was significantly greater in patients with the CC genotype than those with the CT and TT genotypes in GGH 401C>T (2.99 [1.16-7.96]).

**Conclusion:**

This study indicates that MTXPG4 concentrations at four weeks after MTX initiation predict treatment response at 24 weeks. Further, patients with the CC genotype in GGH 401C>T achieve higher MTXPG4 concentrations.

**Main Points**

- MTX treatment responders at 24 weeks achieved higher MTXPG4 concentration in erythrocytes at four weeks compared with nonresponders.
- MTXPG4 concentration in erythrocytes at four weeks was associated with GGH401C>T.
- Methotrexate polyglutamate (MTXPG) concentrations in erythrocytes are a potential methotrexate (MTX) treatment response predictor.

## Introduction

Methotrexate (MTX) is the most predominantly used disease-modifying anti-rheumatic drug (DMARD) in the treatment of rheumatoid arthritis (RA). However, wide interpatient variability is observed in MTX treatment response and unpredictable side effect occurrence. The variability remains unclear, but several factors may play roles, including the MTX concentration, intracellular MTX polyglutamation, and genetic polymorphisms associated with MTX pharmacodynamics and pharmacokinetics.^1^^,^^2^ Thus, identifying early biomarkers to predict MTX efficacy is critical for optimizing individualized therapy. MTX rapidly disappears from plasma after administration,^3^ with plasma MTX concentrations becoming undetectable within 24 hours.^4^ Due to its short half-life, the measurement of plasma MTX concentrations is not suitable for MTX response biomarkers in patients with RA.

Methotrexate (MTX) is transported into cells through Solute carrier family 19 member 1 (SLC19A1).^5^ Inside cells, MTX undergoes sequential polyglutamation by folypolyglutamate synthetase (FPGS), forming MTX polyglutamates (MTXPGs),^6^^,^^7^ which are hydrolyzed by γ-glutamyl hydrolase (GGH).^8^^,9^ Long-chain polyglutamates (MTXPG 4–7) are more stable in cells and exert stronger antifolate effects compared with short-chain polyglutamates.^10^ These metabolites have been proposed as potential biomarkers for both efficacy and toxicity. Previous studies demonstrated associations between MTXPGs and treatment outcome.^11^^-^^16^ However, most studies assessed total MTXPGs at relatively late stages (≥12 weeks), providing limited insight into early prediction of response. The specific role of individual MTXPGs, especially MTXPG4 at the early treatment phase, has not been fully explored. In addition, the influence of genetic polymorphisms in MTX-metabolizing enzymes on early MTXPG accumulation remains unclear.

It is generally believed that MTXPGs with a higher number of glutamate residues may have greater intracellular persistence and potentially stronger anti-inflammatory effects, although this has not been fully established. Based on this concept, we focused on MTXPG4, the longest-chain polyglutamates detectable in erythrocytes in this study. Therefore, this study aimed to determine whether erythrocyte MTXPG4 concentrations measured at four weeks can predict treatment response at 24 weeks. Additionally, we sought to investigate the association between MTXPG4 levels and key single nucleotide polymorphisms (SNPs) involved in MTX metabolism.

## Materials and Methods

### Patients

This retrospective study included patients with RA who initiated MTX treatment from May 2016 to December 2021 at the Shinko Hospital and had been administered MTX continuously for 24 weeks or more.

All the patients met the American College of Rheumatology classification criteria.^17^ Eligible patients had a Disease Activity Score in 28 joints defined as a C-reactive protein (DAS28-CRP) ≥2.7 at MTX treatment initiation.^18^ This study excluded patients who were treated with biological and targeted synthetic DMARDs (b/tsDMARDs) at baseline. In addition, patients who discontinued MTX during the follow-up period due to adverse events were excluded from the final analysis. Eight patients experienced adverse events, including shingles, pneumonia, loss of appetite, or general malaise, and withdrew from the study. These adverse events occurred at MTX doses ranging from 4 mg to 14 mg per week.

Methotrexate (MTX) treatment had been administered at initial doses of 4-8 mg per week. The MTX dose was made at the physician’s discretion and was based on both treatment response and side effects considerations. Among the nine non-responders, five patients did not have their MTX dose increased beyond 8 mg/week. The reasons included adverse effects (elevated liver enzymes, stomatitis, malaise, or abdominal pain) and patient refusal related to ongoing alcohol consumption. Further, each physician added the administration of other medications, including folic acid, prednisolone, and conventional synthetic DMARDs (csDMARDs), when necessary. In this study, concomitant therapy with csDMARDs, was not a criterion for exclusion. The use of NSAIDs was permitted during the study at the discretion of the attending physician. However, patients were excluded from the analysis where MTX efficacy was evaluated if they were receiving b/tsDMARDs or discontinuing MTX treatment because of side effects in the follow-up period. The examination period of this study was 24 weeks after MTX treatment initiation. All patients initially signed written consent for study participation. The Ethics Committee of the Shinko Hospital approved the study protocol (approval number: 2302, Date: April 25, 2023).

### Clinical Data

Patient demographics and clinical data were obtained from each patient’s medical records, including age, sex, body mass index, C-reactive protein (CRP) level, DAS28-CRP, red blood cells, estimated glomerular filtration rate, creatinine, albumin, MTX starting dose, and combined administration of folic acid and prednisolone. In cases of lacking laboratory data, we used the values recorded on the nearest test day within one month before or after the day.

### Assessment of Disease Activity and MTX Efficacy

DAS28-CRP was considered an indicator of disease activity. DAS28-CRP was calculated using the swollen joint count, the tender joint count from among 28 joints, the global assessment of health (measured using a 100-point visual analog scale [VAS]), and CRP.^19^

MTX treatment response was assessed by EULAR response criteria, considering the differences between the DAS28-CRP at initiation of MTX treatment and 24 weeks after treatment.^20^ According to the EULAR-CRP response criteria, we classified the RA patients as responders (good or moderate response) and non-responders (no response).

### Blood Sampling

In routine laboratory tests, 2 mL of whole blood was collected into ethylenediaminetetraacetic acid (EDTA) containing tubes at each visit. The remaining blood samples were utilized for MTXPG measurement and genotyping in erythrocytes.

### Measurement of Methotrexate Polyglutamate Concentrations in Erythrocytes

The EDTA tube was centrifuged at 3000 rpm for 5 minutes to collect 200 µL of erythrocytes. The resulting erythrocytes were stored at −80°C until use. Each sample of MTXPGs (MTXPG1, MTXPG2, MTXPG3, and MTXPG4) was measured utilizing a liquid chromatography-tandem mass spectrometry (LC-MS/MS)-based assay as previously described.^21^ MTXPG5 was scarcely detected throughout the 24 weeks. Each MTXPG and the total MTXPGs were used in the analysis.

### Genotyping

Single nucleotide polymorphisms (SNPs) related to the MTXPG concentrations, SLC19A1 80G>A, FPGS 64A>G, FPGS 192T>C, GGH 452C>T, and GGH 401C>T were genotyped in all patients.

DNA was extracted using the Quick Gene DNA whole blood kit S (Kurabo Industries Ltd) from peripheral blood samples collected from each patient. DNA extraction procedures were conducted according to the manufacturer’s instructions.

Genotyping of alleles of all SNPs was performed utilizing the TaqMan SNP Genotyping Assay from Applied Biosystems (Foster City, CA, USA) with fluorogenic binding probes. Polymerase chain reaction (PCR) amplification with the real-time PCR method was conducted according to the manufacturer’s instructions.

### Statistical Analysis

The comparison and proportions between groups were tested with the Wilcoxon rank sum test, Student’s *t*-test, Fisher’s exact test, and χ^2^ test, following the variable type. Tests for Hardy–Weinberg equilibrium (HWE) were performed using the χ^2^ test to detect potential genotyping errors within the study population. Cut-off MTXPG concentrations with optimal sensitivity and specificity for predicting the responder at 24 weeks were determined using receiver operating characteristic (ROC) curves.

Simple linear regression analysis was conducted to evaluate correlations between MTXPG4 concentrations at four weeks and genetic polymorphisms of the five SNPs, which were further assessed using the multivariate logistic regression model, calculated odds ratios (ORs) for MTXPG4 of ≥0.46 nmol/L at four weeks, and 95% CIs. A forward–backward stepwise selection was utilized. All SNPs were entered together into a multivariable logistic model and then removed until all retained variables demonstrated a *P*-value of <.1. We classified all polymorphisms into two groups to examine the association of mutant alleles with MTXPG4 concentrations at four weeks (wild-type homozygous versus heterozygous and mutant-type homozygous, wild-type homozygous and heterozygous versus mutant-type homozygous). JMP version 10 (SAS Institute Inc; Cary, NC, USA) was used for statistical analysis.

## Results

### Patient Characteristics

**Figure 1.**
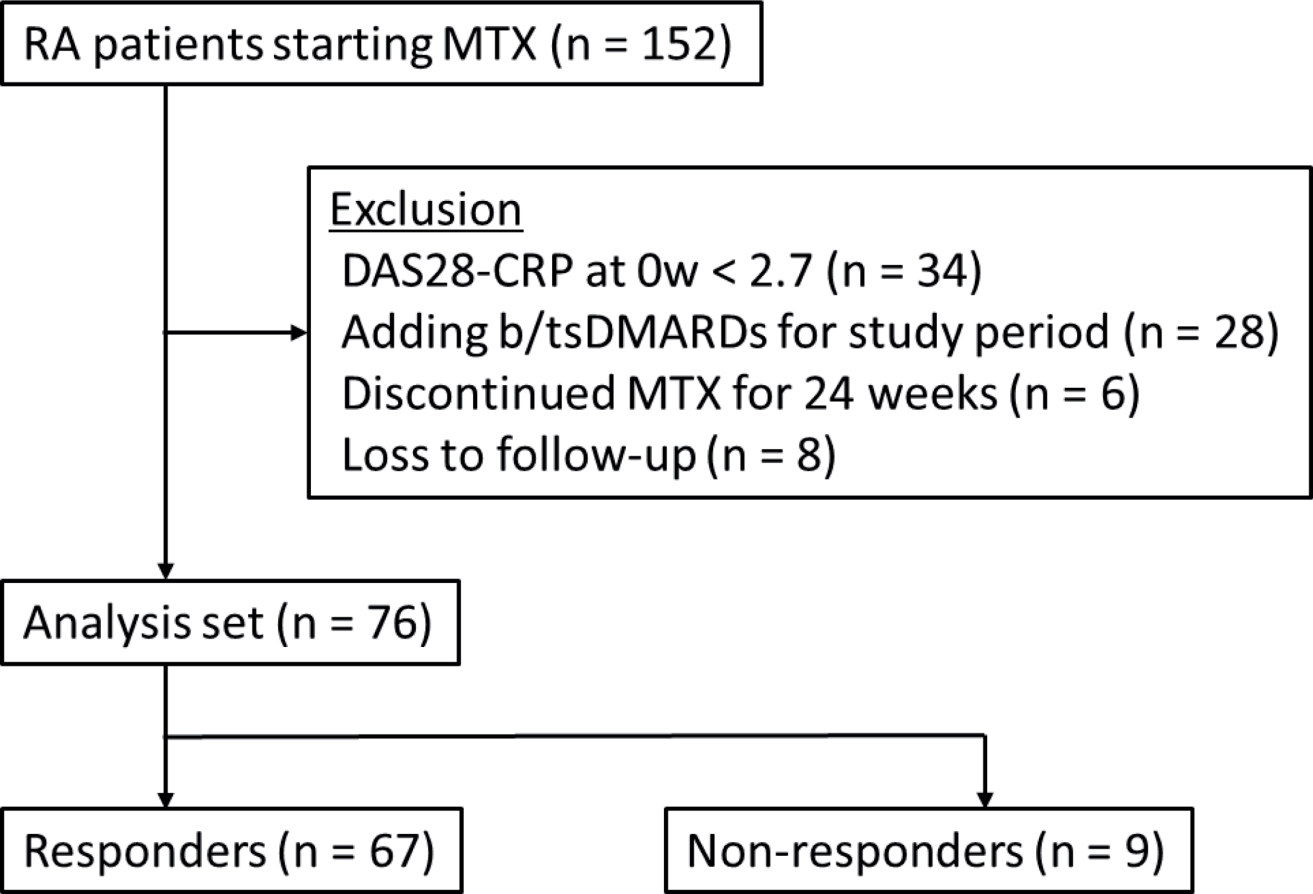
Flowchart of patient enrollment for efficacy analysis. Patients with rheumatoid arthritis (RA) who initiated methotrexate (MTX) treatment between May 2016 and December 2021 were screened. Of the 152 patients initially considered, those with DAS28-CRP <2.7 at baseline (n = 34), who added biologic or targeted synthetic disease-modifying antirheumatic drugs (b/tsDMARDs) during the study period (n = 28), who discontinued MTX before 24 weeks (n = 6), or who were lost to follow-up (n = 8) were excluded. The remaining 76 patients were classified into responders (n = 67) and non-responders (n = 9) based on their treatment response at 24 weeks. RA, rheumatoid arthritis; MTX, methotrexate; DAS28-CRP, Disease Activity Score for 28 joints based on C-reactive protein; b/tsDMARDs, biologic and targeted synthetic disease-modifying anti-rheumatic drugs.

This study enrolled 76 patients with RA (Figure 1). Table 1 shows the patient’s baseline clinical characteristics. Of the participants, 67 were MTX treatment responders at 24 weeks and nine were non-responders. Significantly more females were present in the responders than in the non-responders (52/67 [77.6%] versus 4/9 [44.4%], *P* = .048). DAS28-CRP (median [interquartile range]) at MTX treatment initiation was significantly greater in the responders than in the non-responders (3.90 [3.46-4.91] versus 3.00 [2.91-3.91], *P* = .006). Other parameters, including MTX starting dose, demonstrated no significant differences between the responders and the non-responders.

### Pharmacokinetics of Methotrexate Polyglutamate in Erythrocytes and Disease Activity

**Figure 2.**
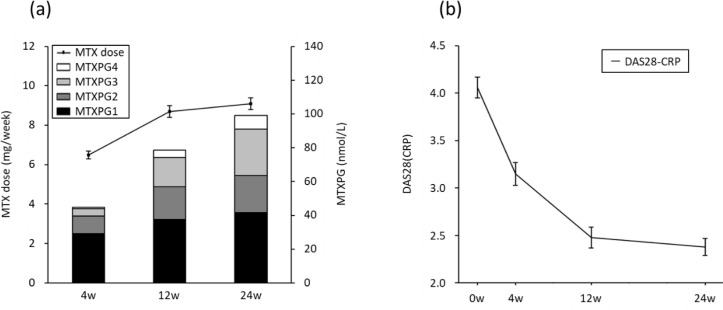
Pharmacokinetics of methotrexate polyglutamates (MTXPGs) concentrations and DAS28-CRP. (a) Mean concentrations of each MTXPG in erythrocyte and MTX dose at 4, 12, and 24 weeks after MTX administration. (b) Mean DAS28-CRP at baseline, 4, 12, and 24 weeks after MTX administration. Data are presented as mean ± standard error. MTX, methotrexate; MTXPG, methotrexate polyglutamate; DAS28-CRP, Disease Activity Score for 28 joints based on CRP level.

Figure 2A illustrates the time course of MTX dose and MTXPG concentrations from four weeks to 24 weeks. The mean MTX dose was increased with time to 6.5 mg/week at four weeks, 8.7 mg/week at 12 weeks, and 9.1 mg/week at 24 weeks. Total MTXPG concentrations steadily increased with MTX dose elevation until 24 weeks. The main fractions of MTXPGs were MTXPG1 and MTXPG2, followed by MTXPG3 and MTXPG4. Figure 2B illustrates the over-time course of DAS28-CRP from baseline to 24 weeks. The mean DAS28-CRP lowered from 4.06 at baseline to 3.15, 2.48, and 2.38 at 4, 12, and 24 weeks, respectively (*P* < .05 at all time points compared to the baseline).

### Association of Methotrexate Polyglutamate Concentrations with Treatment Response

**Figure 3.**
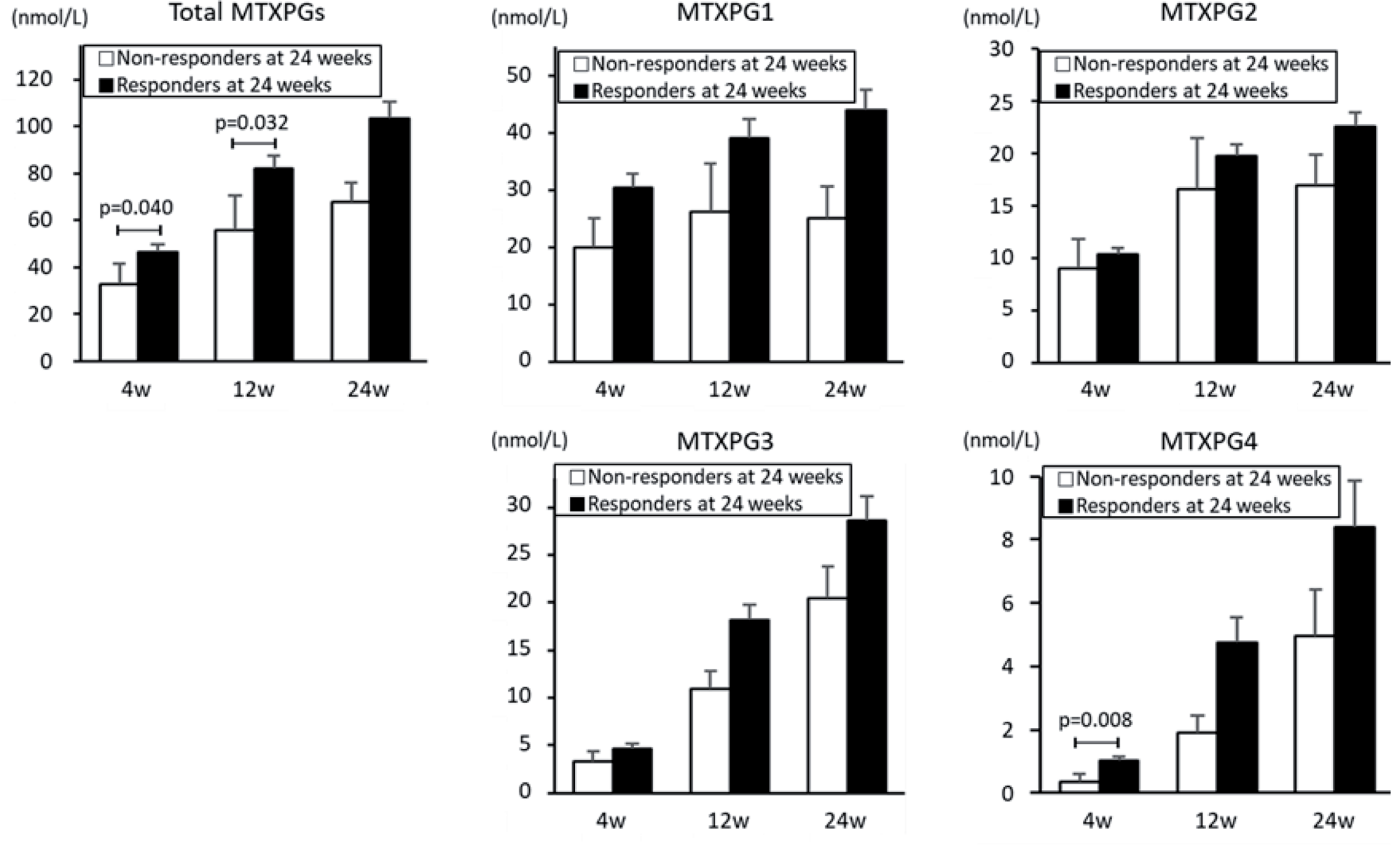
Mean concentration of each methotrexate polyglutamate (MTXPG) at 4, 12, and 24 weeks MTX treatment in responders and non-responders at 24 weeks. Of the 76 patients with RA, 67 were MTX treatment responders and nine were non-responders at 24 weeks. *P*-values for MTXPG concentration between responders and non-responders were assessed with the Wilcoxon rank sum test. Error bars show the standard error. MTXPG, methotrexate polyglutamate.

Figure 3 illustrates MTXPG concentrations with treatment response at 24 weeks. Total MTXPG concentrations at four and 12 weeks and MTXPG4 concentrations at four weeks were significantly greater in responders than in non-responders (total MTXPGs at 4 weeks: 46.6 nmol/L versus 32.6 nmol/L, *P*  = .040; total MTXPGs at 12 weeks: 81.9 nmol/L versus 55.6 nmol/L, *P* = .032; MTXPG4 at four weeks: 1.0 nmol/L versus 0.3 nmol/L, *P* = .008).

Weekly MTX dose at four weeks (mean ± standard error) was significantly greater in responders than in non-responders (6.6 ± 0.2 versus 5.3 ± 0.3 mg/week, *P* = .025).

Furthermore, total MTXPGs concentrations at 12 weeks and MTXPG4 concentrations at four weeks, which were adjusted per 1 mg of weekly MTX dose, were also significantly higher in responders than non-responders: total MTXPGs at 12 weeks, 9.17 nmol/L/mg vs. 9.08 nmol/L/mg, *P* = .049; MTXPG4 at four weeks, 0.15 nmol/L/mg vs. 0.08 nmol/L/mg, *P* = .020. Total MTXPGs concentrations at four weeks, adjusted per 1 mg of weekly MTX dose, were not significantly different between responders and non-responders: total MTXPGs at four weeks, 7.11 nmol/L/mg vs. 6.53 nmol/L/mg, *P* = .096.

### Correlation Between Methotrexate Polyglutamate Concentrations and MTX Efficacy at 24 Weeks

**Figure 4.**
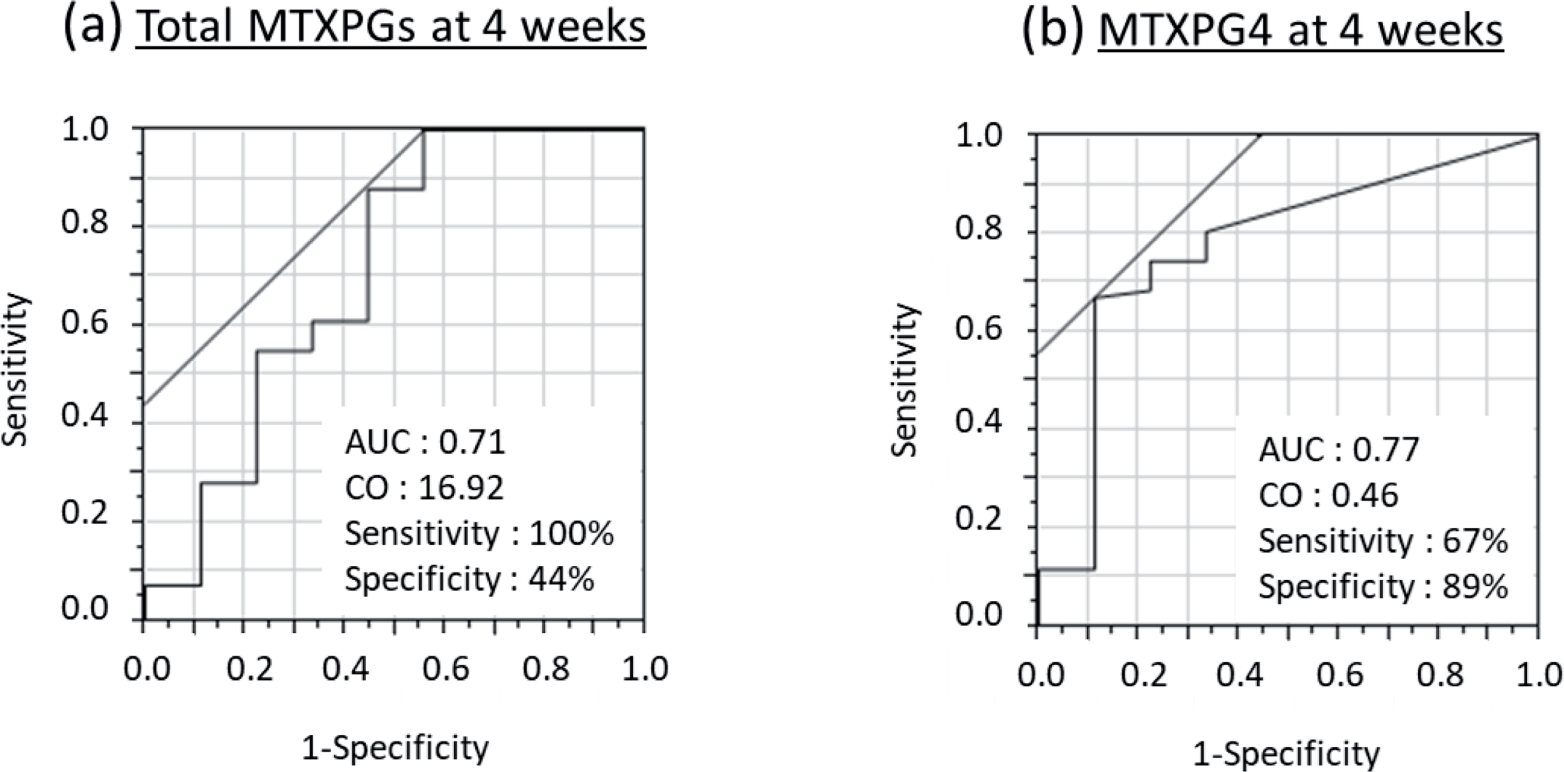
ROC curve for total methotrexate polyglutamates (MTXPGs) and MTXPG4 at four weeks to detect MTX treatment responders at 24 weeks. (a) ROC curve of total MTXPGs at four weeks. (b) ROC curve of MTXPG4 at 4 weeks. MTXPG, methotrexate polyglutamate; AUC: area under the curve; CO, cut-off concentration.

The optimal cut-off values of the total MTXPG and MTXPG4 concentrations at four weeks were investigated for detecting MTX responders at 24 weeks. ROC analyses for detecting MTX responders at 24 weeks using total MTXPGs at four weeks revealed an area under the curve (AUC) of 0.71 with a sensitivity of 100% and specificity of 44%, at the cut-off level of 16.92 nmol/L. MTXPG4 concentrations at four weeks revealed an AUC of 0.77, with sensitivity and specificity of 67% and 89%, respectively, at the cut-off level of 0.46 nmol/L. (Figure 4).

### Clinical Characteristics Affecting MTXPG4 Concentrations at Four Weeks

Based on the ROC analysis, we investigated the factors that affected MTXPG4 concentrations at four weeks. Of the 76 patients categorized based on the MTXPG4 concentrations of 0.46 nmol/L at four weeks, 30 patients demonstrated MTXPG4 of <0.46 nmol/L and 46 patients showed MTXPG4 of ≥0.46 nmol/L at four weeks (Table 2). The median MTX dose at four weeks in patients with MTXPG4 of <0.46 nmol/L was comparable to those having MTXPG4 of ≥0.46 nmol/L (6.0 [6.0-6.5] mg/week versus 6.0 [6.0-8.0] mg/week, *P* = .414). Other clinical parameters at baseline, including MTX dose, exhibited no statistically significant differences between patients with MTXPG4 of <0.46 nmol/L and those with MTXPG4 of ≥0.46 nmol/L at four weeks.

### Genotype Affecting MTXPG4 Concentrations at Four Weeks

To examine the association between the MTXPG4 concentrations in erythrocytes at four weeks and gene polymorphisms of enzymes associated with intracellular MTX metabolism, we then examined polymorphisms of SLC19A1, FPGS, and GGH. In our study, the AA genotype in FPGS 64A>G and the TT genotype in GGH 452C>T were not observed. Table 3 presents univariate and multivariate analyses. The frequencies of all genotypes were congruent with Hardy–Weinberg equilibrium. In this study, only one patient had the TT genotype in GGH 401C>T; hence, we combined CT and TT genotypes as a group of T alleles. Univariate analysis revealed a significantly higher rate of patients achieving MTXPG4 of ≥0.46 nmol/L at four weeks in individuals with CC genotype in GGH 401C>T than in those with CT and TT genotypes in GGH 401C>T (32 of 46 [69.6%] patients with CC genotype versus 14 of 46 [30.4%] patients with CT and TT genotypes, *P* = .032). The crude OR in patients with the CC genotype in GGH 401C>T was significantly greater than in those with the CT and TT genotypes in GGH 401C>T (2.99 [1.15-7.78]).

Multivariate analysis revealed a significantly greater adjusted OR of patients achieving MTXPG4 of ≥0.46 nmol/L at four weeks in individuals with the CC genotype in GGH 401C>T than in those with the CT and TT genotypes (2.99 [1.16-7.96]).

## Discussion

This study demonstrated that erythrocyte MTXPG4 concentrations at four weeks were significantly involved in treatment response at 24 weeks in patients with RA. ROC analysis identified a cut-off value of 0.46 nmol/L, showing favorable discriminative ability (AUC 0.77). Importantly, patients with the CC genotype in GGH 401C>T achieved higher MTXPG4 levels at week four, suggesting a pharmacogenetic determinant of early intracellular MTX retention.

Compared with previous studies, which focused on total MTXPG concentrations or pooled MTXPG3-5 at later time points (≥12 weeks),^15^^,^^16^ our study uniquely highlights MTXPG4 as an independent biomarker measurable at an early phase (at four weeks). Early identification of responders is clinically meaningful, as it may guide timely therapeutic adjustments and reduce unnecessary delays in achieving disease control. To our knowledge, this is the first study to highlight MTXPG4 concentration at four weeks as a possible biomarker for MTX efficacy.

In addition, we analyzed SNPs in genes associated with MTX metabolism, including SLC19A1, FPGS, and GGH. While previous reports have linked these genetic polymorphisms to MTXPG levels,^12^^,^^22^^,^^23^ our study is the first to identify a significant association between the CC genotype of GGH 401C>T and MTXPG4 concentrations at four weeks. GGH hydrolyzes MTXPGs to shorter-chain forms. The CC genotype of GGH 401C>T has been associated with lower GGH enzymatic activity, leading to decreased hydrolysis and accumulation of longer-chain polyglutamates, including MTXPG4. This may explain the higher MTXPG4 concentrations observed in CC carriers.^22^^,^^24^ This supports a mechanistic explanation for interindividual variability in MTX metabolism and treatment efficacy.

Our study has several limitations. First, this was a retrospective study with treatment modifications at the physician’s discretion, and particularly, the potential influence of MTX dose escalation between week four and week 24 was not systematically evaluated. Although a higher MTX dose could lead to higher MTXPG concentrations, responders continued to show higher MTXPG levels even after adjustment for MTX dose, suggesting that MTXPG concentration itself is an important determinant of treatment response. Moreover, the addition of csDMARDs during treatment may have contributed to clinical remission. However, there was no statistically significant difference in the proportion of csDMARD users between the responders and non-responders in our study. Second, only selected SNPs were analyzed, and other potentially relevant variants were not examined. Third, the number of non-responders in this study was small (n = 9), which inevitably limited the statistical power and reliability of subgroup analyses. A post hoc power analysis for the primary outcome yielded a power of 46%, indicating limited sensitivity to detect true differences between groups. This low power may have contributed to the extreme results observed in the ROC analysis, particularly for total MTXPGs, which showed high sensitivity but low specificity. These findings should therefore be interpreted with caution and considered exploratory rather than definitive. Further validation in larger, prospectively designed cohorts is warranted to confirm the utility of MTXPG concentrations—especially MTXPG4—as early predictors of treatment response.

In conclusion, our study demonstrates that MTXPG4 concentration at 4 weeks after MTX initiation is a potential early biomarker for predicting treatment response at 24 weeks. Furthermore, genetic variation in GGH 401C>T may influence MTXPG4 levels, underscoring the importance of integrating pharmacogenomics into individualized MTX therapy.

## Figures and Tables

**Table 1.** Baseline Characteristics of Patients with RA

Characteristics		**Treatment Response at 24 Weeks**		*P*-value
	Total (n = 76)	Responders (n = 67)	Non-responders (n = 9)	
Age, years, mean (SD)	63.3 (12.6)	62.9 (12.7)	65.9 (11.5)	.459
Female, n (%)	56 (73.7)	52 (77.6)	4 (44.4)	.048
BMI, kg/m2, median (IQR)	22.0 (19.3-24.6)	22.0 (18.7-24.2)	24.4 (19.5-26.8)	.368
CRP, mg/dL, median (IQR)	1.14 (0.37-2.76)	1.19 (0.39-2.89)	0.60 (0.23-1.49)	.207
DAS28-CRP, median (IQR)	3.81 (3.31-4.63)	3.90 (3.46-4.91)	3.00 (2.91-3.91)	<.01
RBC, 106/µL, mean (SD)	4.31 (0.50)	4.31 (0.49)	4.33 (0.66)	.917
eGFR, mL/min, median (IQR)	77.4 (66.1-85.5)	79.2 (66.4-86.2)	69.7 (62.0 81.0)	.133
Cr, mg/dL, mean (SD)	0.67 (0.15)	0.65 (0.13)	0.78 (0.20)	.060
ALB, g/dl, median (IQR)	3.9 (3.6-4.2)	3.8 (3.6-4.1)	4.1 (3.8-4.2)	.121
MTX starting dose, mg/week, median (IQR)	6 (6-6)	6 (6-6)	6 (5-6)	.286
csDMARDs, n (%)	30 (39.5)	26 (38.8)	4 (44.4)	.733
Folic acid, n (%)	58 (76.3)	51 (76.1)	7 (77.8)	1.000
Weekly Folic acid dose, mg, median (IQR)	5.0 (5.0-5.0)	5.0 (5.0-5.0)	5.0 (5.0-5.0)	.711
Combined usage of prednisolone, n (%)	23 (30.3)	22 (32.8)	1 (2.2)	.263
Daily prednisolone dose, mg, median (IQR)	5 (3-5)	5 (3-5.3)	4	.528

*P*-values for comparison between responders and non-responders at 24 weeks were assessed with the Wilcoxon rank sum test, Student’s *t*-test, or Fisher’s exact test.

ALB, albumin; BMI, body mass index; Cr, creatinine; CRP, C-reactive protein; csDMARDs, conventional synthetic disease-modifying anti-rheumatic drugs; DAS28-CRP, Disease Activity Score for 28 joints based on CRP level; eGFR, estimated glomerular filtration rate; IQR, interquartile range; MTX, methotrexate; N, number of patients; RA, rheumatoid arthritis; RBC, red blood cell; SD, standard deviation.

**Table 2.** Baseline Clinical Characteristics of Patients Per MTXPG4 Concentrations (<0.46 or ≥0.46) at Four Weeks

Characteristics	**MTXPG4 Concentration at Four Weeks**		*P*-value
	<0.46 nmol/L (n = 30)	≥0.46nmol/L (n = 46)	
Age, years, mean (SD)	62.0 (14.4)	64.1 (11.3)	.516
Female, n (%)	20 (66.7)	36 (78.3)	.262
BMI, kg/m^2^, median (IQR)	22.3 (18.6-26.2)	21.5 (19.8-23.9)	.441
CRP, mg/dL, median (IQR)	0.97 (0.31-2.94)	1.14 (0.43-2.73)	.614
DAS28-CRP, median (IQR)	3.70 (3.06-4.56)	4.04 (3.44-4.98)	.162
RBC, 106/µL, mean (SD)	4.42 (0.53)	4.25 (0.48)	.149
eGFR, mL/min, median (IQR)	80.2 (66.0-90.2)	75.3 (66.1-82.2)	.249
Cr, mg/dL, mean (SD)	0.67 (0.18)	0.67 (0.12)	.976
ALB, g/dl, median (IQR)	4.0 (3.7-4.2)	3.8 (3.6-4.1)	.209
MTX dose at 4w, mg/week, median (IQR)	6.0 (6.0-6.5)	6.0 (6.0-8.0)	.414
Folic acid at 4w, n (%)	26 (86.7)	34 (73.9)	.183
Weekly Folic acid dose at 4w, mg, median (IQR)	5.0 (5.0-5.0)	5.0 (5.0-5.0)	.253
Combined usage of prednisolone at 4w, n (%)	12 (40.0)	22 (48.0)	.502
Daily prednisolone dose at 4w, mg, median (IQR)	5.0 (3.3-5.0)	5.0 (4.1-8.0)	.378

*P*-values for comparison between the groups with <0.46 and ≥0.46 of MTXPG4 concentrations at four weeks were assessed with the Wilcoxon rank sum test, Student’s *t*-test, or Fisher’s exact test.

ALB, albumin; BMI, body mass index; Cr, creatinine; CRP, C-reactive protein; DAS28-CRP, Disease Activity Score for 28 joints based on CRP level; eGFR, estimated glomerular filtration rate; IQR, interquartile range; MTX, methotrexate; N, number of patients; RBC, red blood cell; SD: standard deviation.

**Table 3.** Univariate and Multivariate Analyses for Genetic Factors Related to MTXPG4 Concentrations at Four Weeks

			**MTXPG4 Concentration at Four Weeks, n (%)**			***P*** **-value**
		**Total (n = 76)**	**<0.46 (n = 30)**	**≥0.46 (n = 46)**	**OR (95% CI)**	
Univariate						
SLC19A1 80G>A	GG	15 (19.7)	5 (16.7)	10 (21.7)	1.39 (0.42-4.56)	.828
	GA	33 (43.4)	13 (43.3)	20 (43.5)	1.01 (0.40-2.54)	
	AA	28 (36.8)	12 (40.0)	16 (34.8)	0.80 (0.31-2.07)	
FPGS 64A>G	AG	6 (7.9)	2 (6.7)	4 (8.7)	1.33 (0.23-7.78)	1.000
	GG	70 (92.1)	28 (93.3)	42 (91.3)	0.75 (0.13-4.37)	
FPGS 192T>C	TT	9 (11.8)	2 (6.7)	7 (15.2)	2.51 (0.49-13.02)	.405
	TC	37 (48.7)	17 (56.7)	20 (43.5)	0.59 (0.23-1.49)	
	CC	30 (39.5)	11 (36.7)	19 (41.3)	1.22 (0.47-3.13)	
GGH 452C>T	CC	65 (85.5)	23 (76.7)	42 (91.3)	3.20 (0.85-12.08)	.100
	CT	11 (14.5)	7 (23.3)	4 (8.7)	0.31 (0.08-1.18)	
GGH 401C>T	CC	45 (59.2)	13 (43.3)	32 (69.6)	2.99 (1.15-7.78)	.032*
	CT/TT	31 (40.8)	17 (56.7)	14 (30.4)	0.33 (0.13-0.87)	
Multivariate						
MTX dose at four weeks, mg/week, mean (SD)		6.5 (1.8)	6.3 (1.6)	6.6 (1.9)	－	.491
SLC19A1 80G>A	GG	15 (19.7)	5 (16.7)	10 (21.7)	－	.817
	GA/AA	61 (80.3)	25 (83.3)	36 (78.3)		
	GG/GA	48 (63.2)	18 (60.0)	30 (65.2)	－	.786
	AA	28 (36.8)	12 (40.0)	16 (34.8)		
FPGS 64A>G	AG	6 (7.9)	2 (6.7)	4 (8.7)	－	.786
	GG	70 (92.1)	28 (93.3)	42 (91.3)		
FPGS 192T>C	TT	9 (11.8)	2 (6.7)	7 (15.2)	－	.388
	TC/CC	67 (88.2)	28 (93.3)	39 (84.8)		
	TT/TC	46 (60.5)	19 (63.3)	27 (58.7)	－	.605
	CC	30 (39.5)	11 (36.7)	19 (41.3)		
GGH 452C>T	CC	65 (85.5)	23 (76.7)	42 (91.3)	－	.371
	CT	11 (14.5)	7 (23.3)	4 (8.7)		
GGH 401C>T	CC	45 (59.2)	13 (43.3)	32 (69.6)	2.99 (1.16-7.96)	.025*
	CT/TT	31 (40.8)	17 (56.7)	14 (30.4)		

*P*-values for comparisons between the groups with MTXPG4 concentrations of <.46 and ≥.46 at four weeks were assessed with Fisher’s exact test. *P*-values were obtained by multiple logistic regression analysis using a step-wise procedure.

95% CI, 95% confidence interval; FPGS, folylpolyglutamate synthase; GGH, γ-glutamyl hydrolase; MTX, methotrexate; N, number of patients; OR, odds ratio; SLC19A1, reduced folate carrier 1.

## Data Availability

The data that support the findings of this study are available on request from the corresponding author.
